# Untargeted Metabolomics Based on UPLC-Q-Exactive-Orbitrap-MS/MS Revealed the Differences and Correlations between Different Parts of the Root of *Paeonia lactiflora* Pall

**DOI:** 10.3390/molecules29050992

**Published:** 2024-02-24

**Authors:** Jiahui Lv, Qianqian Du, Suying Shi, Mengzhen Ma, Wei Zhang, Dezhu Ge, Lihua Xing, Nianjun Yu

**Affiliations:** 1 School of Pharmacy, Anhui University of Chinese Medicine, Hefei 230012, China; 2 MOE-Anhui Joint Collaborative Innovation Center for Quality Improvement of Anhui Genuine Chinese Medicinal Materials, Hefei 230012, China; 3 Anhui Province Key Laboratory of Research, Development of Chinese Medicine, Hefei 230012, China; 4 Anhui Jiren Pharmaceutical Co., Ltd., Bozhou 236800, China

**Keywords:** *Paeonia lactiflora* Pall., UPLC-MS/MS, untargeted metabolomics, stoichiometry, KEGG pathway analysis

## Abstract

Background: *Paeonia lactiflora* Pall. (PLP) is a plant with excellent ornamental and therapeutic value that can be utilized in traditional Chinese medicine as *Paeoniae Radix* Alba (PRA) and *Paeoniae Radix* Rubra (PRR). PRA must undergo the “peeling” process, which involves removing the cork and a portion of the phloem. PLP’s biological function is strongly linked to its secondary metabolites, and the distribution of metabolites in different regions of the PLP rhizome causes changes in efficacy when PLP is processed into various therapeutic compounds. Methods: The metabolites of the cork (cor), phloem (phl), and xylem (xyl) were examined in the roots of PLP using a metabolomics approach based on UPLC-Q-Exactive-Orbitrap-MS/MS (UPLC-MS/MS), and the differential metabolites were evaluated using multivariate analysis. Results: Significant changes were observed among the cor, phl, and xyl samples. In both positive and negative ion modes, a total of 15,429 peaks were detected and 7366 metabolites were identified. A total of 525 cor-phl differential metabolites, 452 cor-xyl differential metabolites, and 328 phl-xyl differential metabolites were evaluated. Flavonoids, monoterpene glycosides, fatty acids, sugar derivatives, and carbohydrates were among the top 50 dissimilar chemicals. The key divergent metabolic pathways include linoleic acid metabolism, galactose metabolism, ABC transporters, arginine biosynthesis, and flavonoid biosynthesis. Conclusion: The cor, phl, and xyl of PLP roots exhibit significantly different metabolite types and metabolic pathways; therefore, “peeling” may impact the pharmaceutical effect of PLP. This study represents the first metabolomics analysis of the PLP rhizome, laying the groundwork for the isolation and identification of PLP pharmacological activity, as well as the quality evaluation and efficacy exploration of PLP.

## 1. Introduction

*Paeonia lactiflora* Pall., with a rich history of approximately 3000 years, has been mentioned in ancient texts such as the *Book of Songs* and *Shennong Bencao Jing*, marking its significance in traditional Chinese medicine. Its first recorded medicinal use dates back to *Fifty-two Prescriptions*, and today, it is extensively employed in the pharmaceutical field [1]. Pharmacological studies have demonstrated its efficacy in treating a variety of conditions including depression, atherosclerosis, rheumatoid arthritis, liver damage, ulcerative colitis, Parkinson’s disease, and Alzheimer’s disease [2].

Clinically, PLP is utilized in two primary forms: *Paeoniae Radix* Rubra (PRR) and *Paeoniae Radix* Alba (PRA). As per the 2020 edition of the Chinese Pharmacopoeia [3], PRA is the dried medicinal material of the Ranunculaceae family’s *Paeonia lactiflora* Pall., boiled and peeled, known for its blood-nourishing and menstruation-regulating properties, as well as liver-soothing and pain-relieving effects. PRR, on the other hand, represents the direct use of the dried medicinal material of the plant, effective in clearing heat, cooling blood, breaking blood stasis, and alleviating pain. PLP is composed of three layers from the outside in: cor, phl, and xyl. The “skin” removed during the PRA production process includes the cork and a minimal amount of the phloem, whereas the dried PRA contains portions of both the phloem and xylem. Researchers believe that the pharmacological and pharmacodynamic differences between PRA and PRR stem from their distinct processing methods, which impact the concentration of chemical constituents and thus the efficacy of the medicinal material [1]. Studies have identified significant amounts of benzoic acid and albiflorin in the outer skin of the PLP root rhizome [4,5], with paeoniflorin and albiflorin primarily located in the cork and cortex [6]. Paeoniflorin, serving as a quality marker for both PRA and PRR, is crucial to the quality of PLP medicinal materials [3]. Hence, research into the various parts of the PLP root can deepen our understanding of its chemical composition differences and pharmacological properties, further improving quality control and providing more reliable support for clinical applications.

Recent advancements in metabolomics and chemometrics have offered new perspectives in understanding PLP. Studies using modern instruments such as HPLC, ^1^H-NMR, UPLC-Q/TOF-MS, and HPLC-DAD-ESI-MS have elucidated the chemical composition differences among various peony varieties [7], cultivation regions [8], and different parts of the plant such as the roots, stems, and flowers [9]. Furthermore, research utilizing these methods has aided in analyzing the distribution of eleven chemical components including peony glycosides in the PLP root [10]. However, limitations in the sensitivity, separation, and detection thresholds of instruments such as HPLC and UPLC have restricted the analysis to only a few chemical components, leaving a gap in understanding the full spectrum of chemical variations in the PLP root. Additionally, a detailed exploration of the chemical composition differences among the cork, phloem, and xylem of the PLP root remains unexplored. This research gap forms the basis of this study, which focuses on investigating the chemical component distribution in these specific parts of the PLP root to more comprehensively reveal the chemical characteristics and pharmacological mechanisms of PLP.

The integration of untargeted metabolomics with chemometrics has become an important and valuable tool in various life science research areas in recent years, including biomarker discovery, disease diagnosis, and the quality assessment of food and herbal medicines [11,12]. The strength of untargeted metabolomics lies in its ability to analyze and compare a multitude of complex components without the need for prior knowledge of the material composition or key metabolic products [13]. Chemometric methods can be used to extract crucial information from raw data, revealing potential correlations among a vast array of variables. Tandem mass spectrometry coupled with UPLC-MS/MS is widely regarded as the preferred and effective method for the analysis of traditional Chinese medicine, providing structural fragment information of compounds at different retention times [14]. UPLC-MS/MS not only enables the chemical analysis of traditional Chinese medicine samples but also facilitates the analysis of different parts and sources of plants. Additionally, it allows for a rough estimation of the relative content of components based on signal intensity [15].

In summary, this study, by combining untargeted metabolomics and chemometric methods with UPLC-MS/MS, aims to reveal the metabolic profile differences in various parts of the PLP root and further explore the impact of these differences on the chemical properties and pharmacological mechanisms of PLP. This research not only promises to provide new scientific evidence for the quality control of PLP but also deepens our understanding of this ancient herbal medicine, supporting its broader medical applications. It also offers new perspectives and possibilities for drug development and clinical treatment.

## 2. Results and Discussion

### 2.1. Multivariate Statistical Analysis

In this study, a total of 15,429 substance peaks were detected, with 7366 unique metabolites being identified. Among them, 5109 were identified under ESI+ mode and 2257 were identified under ESI− mode. Notably, no metabolites were detected simultaneously in both ESI+ and ESI− modes, indicating distinct metabolomic profiles captured by each ionization technique. The base peak chromatograms for both positive and negative ion modes are presented in Supplementary Material Figure S1. Multivariate statistical analyses, including principal component analysis (PCA), partial least-squares–discriminant analysis (PLS-DA), and orthogonal partial least-squares–discriminant analysis (OPLS-DA), were applied to the detected substance peaks.

PCA was utilized to understand the overall situation of the metabolites. PLS-DA was employed to distinguish metabolic differences between different groups, and OPLS-DA was used for model refinement, noise reduction, and enhancing analytical power and effectiveness. It emphasized the results provided by PCA and PLS-DA and highlighted the differences both between and within the sample groups. Additionally, seven-fold cross-validation and 200-times response permutation testing (RPT) were conducted to assess whether the models suffered from overfitting.

**Figure 1:**
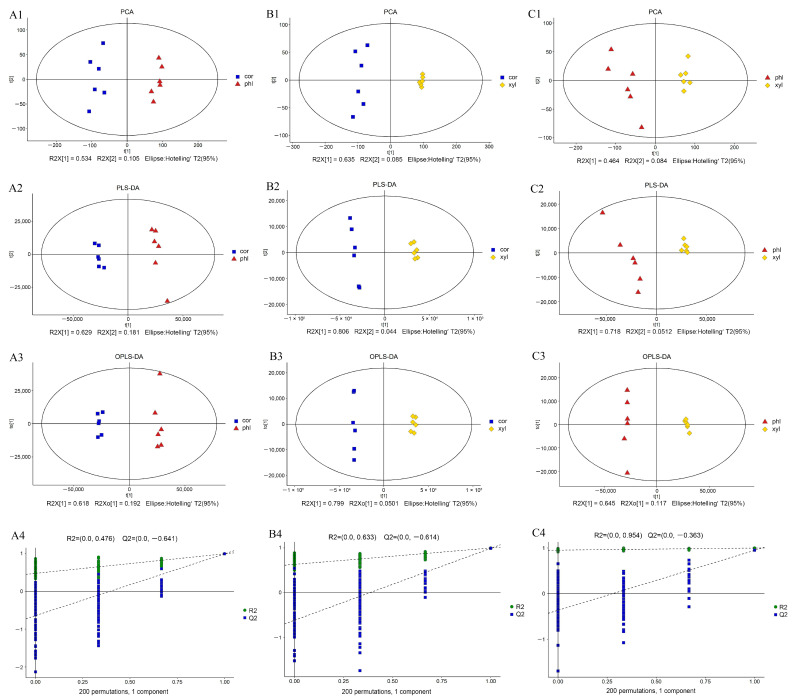
Multivariate analysis among the cor, phl, and xyl groups. (**A**): cor-phl; (**B**): cor-xyl; (**C**): phl-xyl; (**1**): PCA; (**2**): PLS-DA; (**3**): OPLS-DA; (**4**): permutation. R2X (cum): cumulative explained variance in the X-direction; R2Y (cum): cumulative explained variance in the Y-direction; Q2 (cum): cumulative predictive ability of the model; R2 and Q2: parameters of the permutation test, used to evaluate model overfitting.

The model parameters for multivariate statistical analysis among the three groups are shown in Supplementary Material Table S1. The results of the PCA, PLS-DA, and OPLS-DA analyses are presented in Figure 1. The PCA plots demonstrate clear separations between the three groups, with high values of R2X[1] indicating that the first principal component significantly contributes to the explanation of data variability. No obvious outliers were observed, indicating the good statistical consistency of the model (Figure 1(A1–C1)). In the PLS-DA plots, the values of R2X[1] and R2X[2] confirm the differentiation capability of PLS-DA, showcasing its efficiency. The values of parameters R2Y and Q2 were 0.995 and 0.992; 0.998 and 0.993; and 0.998 and 0.944, respectively (Supplementary Material Table S1), indicating the good predictive ability of the PLS-DA model (Figure 1(A2–C2)). The OPLS-DA plots further revealed significant differences between cor, phl, and xyl. The values of R2X[1] and R2X0[1] demonstrated the efficacy of OPLS-DA in capturing key differences between groups (Figure 1(A3–C3)). The 200-times permutation test for cross-validation showed that R2 was close to 1 and Q2 was less than 0, indicating the high adaptability and reliability of the OPLS-DA model in handling complex metabolic data (Figure 1(A4–C4)).

### 2.2. Analysis of Differential Metabolites

**Figure 2:**
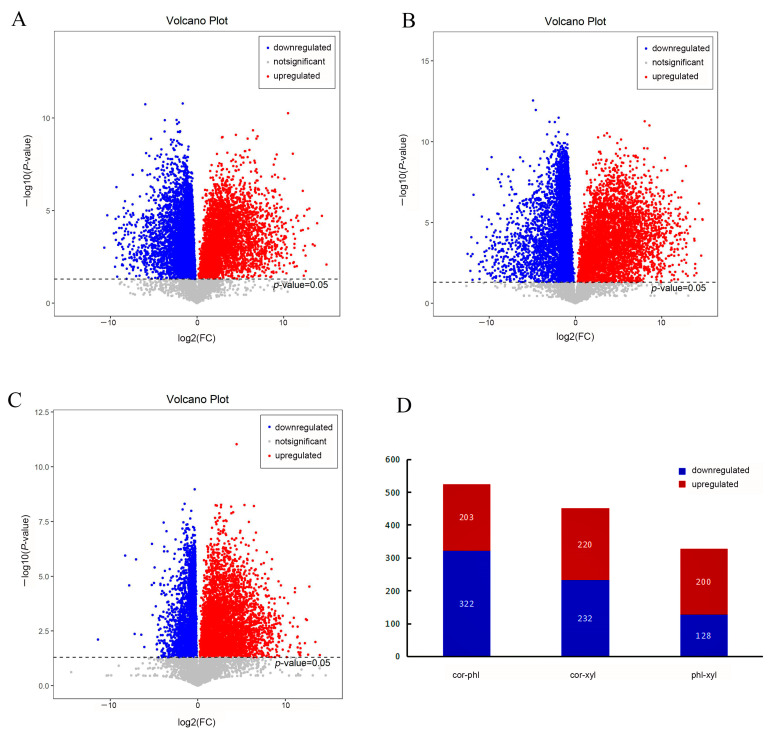
Comparison of differential metabolites among the cor, phl, and xyl groups. (**A**): cor-phl; (**B**): cor-xyl; (**C**): phl-xyl; (**D**): the number of differential metabolites among the groups. Differential metabolites were selected with a threshold of variable importance in projection (VIP) > 1.0, *p*-value < 0.05. Red dots indicate significant upregulation (log2(FC) > 0), blue dots indicate significant downregulation (log2(FC) < 0), and gray dots indicate no significant difference.

Univariate analysis methods such as the Student’s *t*-test and fold change analysis were employed to compare the expression of differential metabolites between the different experimental groups. The fold change (FC) values of the detected substance peaks were converted to log2 (FC), and the *p*-values from the Student’s *t*-test (*p* = 0.05) were converted to −log10 (*p*-value) for the construction of volcano plots (Figure 2).

As indicated in the volcano plots, significant metabolic changes exist between cor and xyl, with the highest number of significantly downregulated metabolite points, suggesting the potential inhibition of key metabolic pathways in cor (Figure 2B). Between phl and xyl, there are fewer significantly downregulated points and more non-significant gray points (Figure 2C), indicating smaller biological differences between these two tissues. Further analysis revealed that the metabolites between phl and xyl show higher significance, and the change magnitude between cor and phl is greater, suggesting more significant metabolite variations between phl and xyl and a larger magnitude of changes between cor and phl.

A total of 525 differential metabolites were identified between the cor and phl groups in the PLP root, with 203 metabolites upregulated and 322 metabolites downregulated. Between cor and xyl, 452 differential metabolites were identified, with 220 upregulated and 232 downregulated. Between phl and xyl, 328 differential metabolites were identified, with 200 upregulated and 128 downregulated (Figure 2D).

### 2.3. Analysis of the Top 50 Differential Metabolites

**Figure 3:**
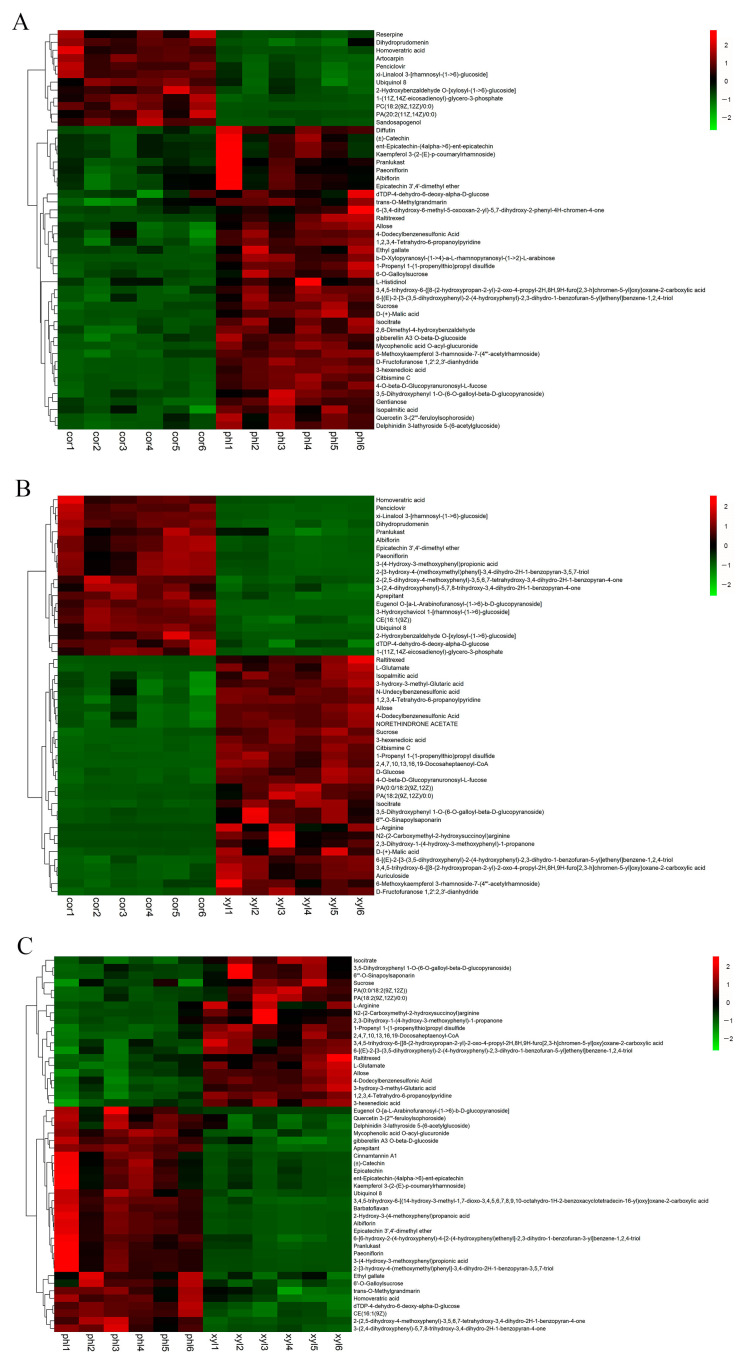
Heatmap of the top 50 differential metabolites among the cor, phl, and xyl groups. (**A**): cor-phl; (**B**): cor-xyl; (**C**): phl-xyl. The horizontal axis represents the sample names, and the vertical axis represents the top 50 differential metabolites. The color gradient from green to red indicates the expression abundance of metabolites from low to high.

**Figure 4:**
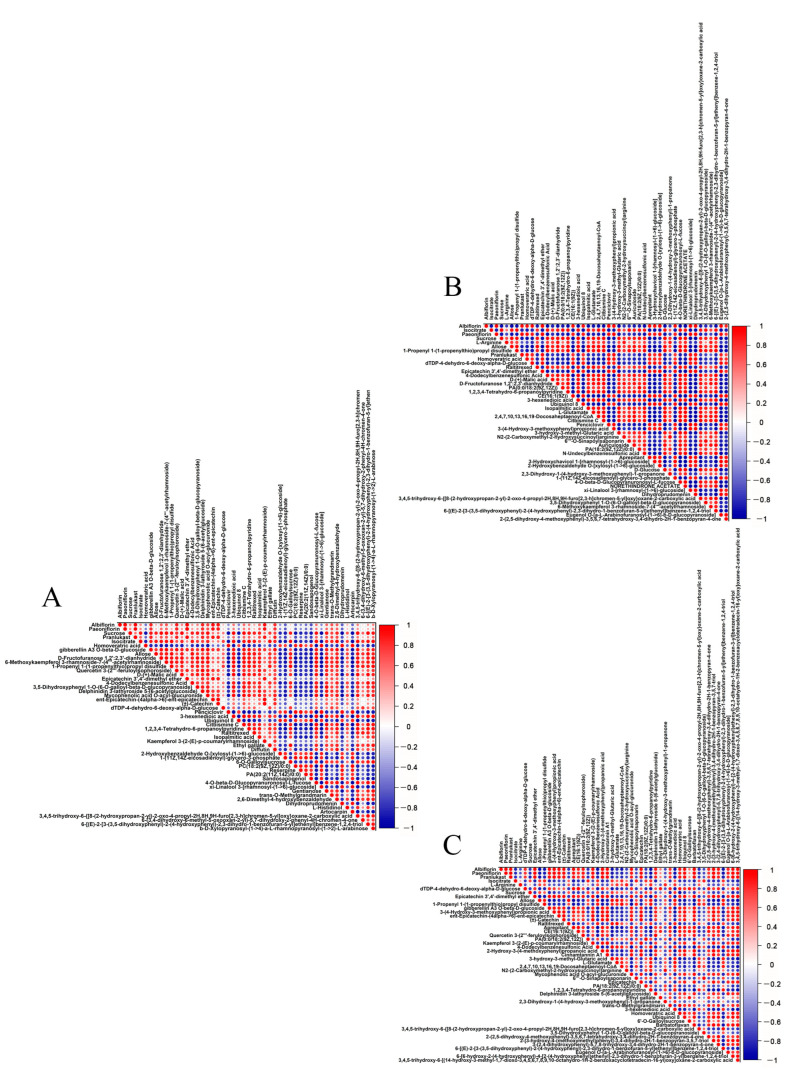
Correlation analysis of top 50 differential metabolites among the cor, phl, and xyl groups. (**A**): cor-phl; (**B**): cor-xyl; (**C**): phl-xyl. The correlation coefficient ranges from −1 to +1, with close to +1 (red) indicating a strong positive correlation, close to −1 (blue) indicating a strong negative correlation, and close to 0 indicating no significant correlation.

To further analyze the expression differences in metabolites among different samples, hierarchical clustering was conducted on all significantly differential metabolites. Heatmaps and correlation analysis plots for the top 50 differential metabolites were created using Origin 2022 software based on VIP values and Pearson product–moment correlation coefficients (Figure 3 and Figure 4). Details of the top 50 differential metabolites can be found in Supplementary Material Table S2.

Through categorization and comparison of the top 50 differential metabolites, 678 pairs of metabolites between cor and phl showed a significant positive correlation, while 423 pairs exhibited a significant negative correlation. Between cor and xyl, there were 648 pairs with a significant positive correlation and 600 pairs with a significant negative correlation. Between phl and xyl, 608 pairs were significantly positively correlated, and 586 pairs were negatively correlated.

The study identified a diverse range of compound categories, including monoterpenoid glycosides, tannins, flavonoids and their glycosides, coumarins, organic acids, amino acids, nucleosides, sugars and their derivatives, and nitrogen-containing heterocyclic compounds. Monoterpenoid glycosides, particularly paeoniflorin and albiflorin, showed consistent positive correlations across cor, phl, and xyl, indicating their stable biological roles in the different tissues of peony roots, such as anti-inflammatory, anti-tumor, and immune regulatory functions [16,17,18]. The high abundance of flavonoids and their glycosides in phl reflects the tissue’s activity in antioxidation and environmental stress resistance [19,20]. The low abundance of coumarin compounds in cor suggests their potential specific roles in the regulation of cork layer growth. Furthermore, the higher abundance of fatty acids in phl and the highest abundance of Ubiquinol 8 in cor indicate cor’s potential function in maintaining cell membrane stability and energy transfer [21,22]. Overall, the levels of primary metabolites (such as amino acids, nucleosides, and sugars) and secondary metabolites (such as monoterpenoid glycosides, flavonoids, coumarins, etc.) were generally higher in phl than in cor, suggesting phl’s advantage in energy metabolism and the synthesis of secondary metabolites.

However, the metabolic differences between phl and xyl showed less regularity, hinting at potential complex metabolic regulatory mechanisms. Further research into these metabolic differences and their roles in plant physiology and adaptability will provide a deeper understanding of the biological functions of peony roots and their mechanisms in responding to environmental changes.

### 2.4. Enrichment Analysis of Metabolic Pathways

**Figure 5:**
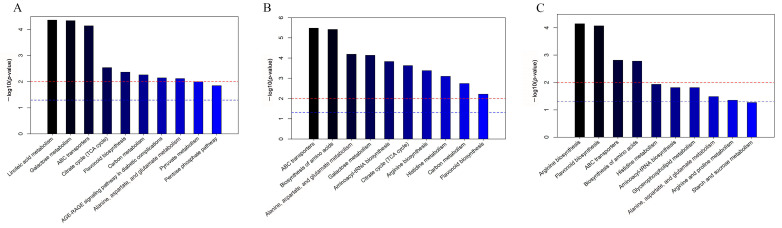
Enrichment graphs of the top 10 metabolic pathways among the cor, phl, and xyl groups. (**A**): cor-phl; (**B**): cor-xyl; (**C**): phl-xyl. The red line indicates a *p*-value of 0.01, and the blue line indicates a *p*-value of 0.05. When the top of a bar exceeds the blue line, the corresponding pathway is significantly enriched.

**Figure 6:**
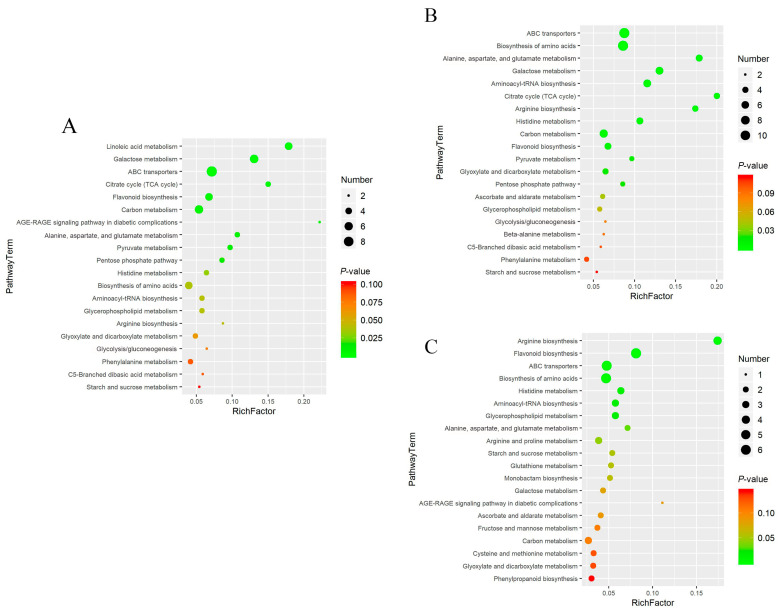
Bubble charts of the top 20 among the cor, phl, and xyl groups. (**A**): cor-phl; (**B**): cor-xyl; (**C**): phl-xyl. The vertical axis represents the metabolic pathways, and the horizontal axis represents the enrichment factor. A larger Rich factor indicates greater enrichment. The color gradient from red to green indicates decreasing *p*-values; larger bubbles indicate a greater number of compounds enriched in the pathway.

Enrichment analysis of metabolic pathways was performed using the Kyoto Encyclopedia of Genes and Genomes (KEGG) IDs of differential compounds to obtain the results of pathway enrichment. A *p*-value threshold of ≤0.05 was established for selecting the top 10 significantly enriched pathways among the differential metabolites (Figure 5). Subsequently, a bubble chart was constructed to graphically represent these pathways (Figure 6).

The results showed that linoleic acid metabolism, galactose metabolism, and ABC transporters were the primary metabolic pathways contributing to the differences between cor and phl in PLP. Arginine biosynthesis and flavonoid biosynthesis were the main metabolic pathways differentiating phl and xyl. It was noted that significant differential metabolites included L-Arginine, a product of the arginine biosynthesis pathway, and Quercetin 3-(2′′′-feruloylsophoroside), Kaempferol 3-(2-(E)-p-coumarylrhamnoside), and 6-Methoxykaempferol 3-rhamnoside-7-(4′′′-acetylrhamnoside), which are products of the flavonoid biosynthesis pathway. These metabolic pathways collectively contribute to the distinctiveness among different parts within PLP.

Linoleic acid, an essential fatty acid in humans, primarily functions in cell membrane synthesis and inflammation regulation [22,23]. Previous studies have reported that linoleic acid can protect the liver, improve liver fibrosis [24,25], and offer cardiac protection in rats with chronic heart failure by modulating its metabolism [26]. It also alleviates lipopolysaccharide-induced acute inflammation and multi-organ damage [23]. Galactose, a vital carbohydrate in cell metabolism, can be directly absorbed by target tissues, promoting energy production, glycosylation, and other metabolic functions [27]. Due to its chemical properties that allow for transmembrane diffusion, galactose is clinically used to generate glycoconjugates for improved drug absorption, as successfully demonstrated in supplementing dopamine for Parkinson’s patients [28]. Regulating galactose metabolism has been shown to alleviate acute liver damage and improve some complications of endotoxemia [29,30]. Earlier studies indicated that fatty acid and sugar metabolites are more abundant in phl, suggesting that PLP might regulate metabolic pathways through phl, thus exerting hepatoprotective and menstrual regulation effects.

L-Arginine, a precursor to proteins and NO, is known as a “vascular scavenger”, acting as a vasodilator and an endogenous anti-atherosclerosis molecule in the cardiovascular system [31,32]. Its significant metabolic roles have been confirmed, as L-Arginine positively influences energy metabolism through various physiological and metabolic mechanisms and can orally delay or prevent progressive renal failure in mice [33]. Flavonoids, as secondary metabolites widely present in plants, play crucial roles in plant growth and resistance to adverse stress [20]. Research shows that stress conditions enhance the expression of flavonoid synthase genes, leading to flavonoid accumulation. These compounds are transported to organelles through various pathways, adjusting plant growth patterns to adapt to the environment [34]. Additionally, the hydroxyl groups in flavonoid structures confer strong antioxidant properties [35]. Flavonoids released from roots can also act as signaling molecules, promoting the colonization and growth of root microorganisms, thereby strengthening plant–microbe symbiosis against environmental stress [36,37]. Cor, phl, and xyl may modulate metabolic pathways through changing metabolites, thereby enabling PLP to exert various pharmacological effects, such as the anticoagulant action of PRR in a rat model of acute blood stasis. Further study into the changes of metabolic biomarkers in PLP and their impact on metabolic pathways is anticipated to provide references for discovering new therapeutic targets and more effective treatment strategies.

## 3. Materials and Methods

### 3.1. Reagents and Materials

Methanol, formic acid, ultra-pure water, and acetonitrile were purchased from ANPEL Laboratory Technologies Inc. in Shanghai. L-2-chlorophenylalanine was obtained from Shanghai HC Biotech Co., Ltd. (Shanghai, China). LysoPC17:0 was sourced from Avanti Polar Lipids, Inc. (Alabaster, AL, USA). All chemical reagents and solvents were of analytical purity or chromatographic grade.

**Figure 7:**
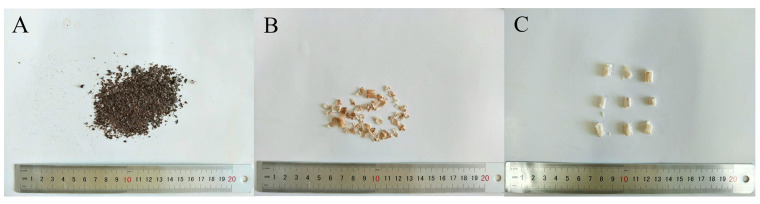
Photos of the samples. (**A**): cor; (**B**): phl; (**C**): xyl.

*Paeonia lactiflora* Pall. specimens were collected in August 2019 from Bozhou, Anhui Province. Professor Yu Nianjun of Anhui University of Traditional Chinese Medicine authenticated the plant, and the botanical specimens were meticulously prepared and archived at the School of Pharmacy, Anhui University of Traditional Chinese Medicine. Samples were obtained from six distinct PLP plants. Cor1, phl1, and xyl1 were isolated from different sections of the same PLP root, and so forth. For a detailed overview of the sample materials, please refer to Figure 7.

### 3.2. Sample Preparation

Approximately 10 mg of powder was weighed and then placed into a 1.5 mL centrifuge tube. Subsequently, 1 mL of 50% ethanol was added, and the total weight of the mixture was measured. The mixture was then ultrasonicated for 30 min. After ultrasonication, the sample was cooled, and any weight loss was compensated for. The sample was then centrifuged for 10 min at 13,000 rpm and 4 °C. A total of 200 μL of the supernatant was aspirated using a syringe and then filtered through a 0.22 μm microporous membrane. If not used immediately, the sample could be stored at −80 °C. Quality control (QC) samples were prepared by combining equal volumes of extracts from all samples. All extraction reagents were pre-cooled at −20 °C before use.

### 3.3. UPLC-MS/MS Analysis

In this study, an ACQUITY ultra-high-performance liquid chromatography system (Waters, Milford, MA, USA) was coupled with a QE high-resolution mass spectrometer (Thermo Fisher Scientific, Waltham, MA, USA). Chromatographic separation was performed using an ACQUITY UPLC BEH C18 column (100 mm × 2.1 mm, 1.7 μm) (Waters, USA). The mobile phases consisted of 0.1% formic acid in water (A) and 0.1% formic acid in acetonitrile (B). The gradient elution was as follows: 0–2 min, 5–20% B; 2–4 min, 20–25% B; 4–9 min, 25–60% B; 9–14 min, 60–100% B; 14–16 min, 100% B; 16–16.1 min, 100–5% B; 16.1–18.1 min, 5% B. The column temperature was maintained at 40 °C with a flow rate of 0.35 mL/min, and the injection volume was 5 μL.

The mass spectrometry scan parameters were set as follows: S-lens RF level, 50; mass range, 100 to 1200 *m*/*z*; full MS resolution, 70,000; MS/MS resolution, 17,500; NCE/stepped NCE was set at 10, 20, and 40 eV. The ESI ion source parameters were set as follows: spray voltage, 3800 V; sheath gas flow rate, 40 for ESI+ and 5 for ESI−; capillary temperature, 320 °C; probe heater temperature, 350 °C; aux gas flow rate, 10. During the mass spectrometry operation, one QC sample was inserted for every six formal samples. The QC samples were used to evaluate the stability of the mass spectrometry platform throughout the experimental process.

### 3.4. Data Preprocessing

Raw data were collected using UNIFI 1.8.1 software. Subsequently, data preprocessing was carried out using Progenesis Ql v2.3 software, developed by Nonlinear Dynamics in Newcastle, UK. The preprocessing steps encompassed baseline filtration, peak identification, integration, retention time correction, peak alignment, and normalization. The primary parameters employed were as follows: precursor tolerance: 5 ppm; product tolerance: 10 ppm; and product ion threshold: 5%. For compound qualitative analysis, we relied predominantly on the Human Metabolome Database (HMDB), Lipidmaps (v2.3), and the METLIN database. Experimental mass spectrometry data were aligned with known compound information from these databases, encompassing accurate mass, secondary fragment ions, and isotope distribution, among other details.

Within the obtained compound data, ion peaks with group-wide missing values (zero values) exceeding 50% were omitted. Zero values were replaced with half of the minimum value to maintain data integrity. Subsequently, a scoring system was applied to the qualitative identification results, with a maximum score of 60 points. This scoring system comprised three components: first-level mass spectrometry exact mass matching (20 points), second-level mass spectrometry fragment matching (20 points), and isotope distribution matching (20 points). A filtering criterion of 30 points or higher was set, and results scoring below 30 points were deemed uncertain and excluded. Finally, positive and negative ion data were amalgamated into a data matrix table, which encompassed identifiers, *m*/*z* ratios, retention times, ion modes, and metabolite identifications, along with compound classification and fragmentation scores, etc., serving as the foundation for all subsequent analyses.

## 4. Conclusions

This study applied UPLC-MS/MS in conjunction with metabolomics to deeply investigate the metabolic differences in various parts of the PLP root (cor, phl, and xyl) and their potential roles in the biological activities of PLP. The results showed significant metabolic spectrum differences between the different parts and also revealed the distribution characteristics of primary and secondary metabolites in PLP.

Primary metabolism and its metabolites are essential for maintaining the basic life activities of cells [38], with the phl part of the PLP root having the highest level of primary metabolites. This indicates that the vitality of phl is stronger than that of cor and xyl. Secondary metabolism and its metabolites are produced as plants adapt to their environment, helping them to combat adverse conditions, resist pests and diseases, enhance species’ competitive advantage, and provide rich industrial raw materials [39]. They can also be used to discover bioactive components for health care, medicine, and many other industries [40]. The overall level of secondary metabolites in PLP is also higher in phl, with cor having relatively lower content. The differences in the content and types of chemical components among the three parts may be the reason for the differences in the medicinal effects of PRA and PRR. Previous studies have confirmed that the cork contains higher levels of paeoniflorin, paeonol glycosides, and benzoic acid, making it a viable source of effective medicinal compounds [41]. The consideration of whether to retain the cork can be made after further research into the impact of “decorking” on the pharmacological actions of PLP. Our study is the first to reveal the metabolic patterns of compounds in the root parts of PLP from a metabolomics perspective, providing assistance for the rational processing and utilization of PLP and laying a foundation for the further development of PLP’s medicinal effects.

## Data Availability

The datasets generated and analyzed during the course of this study are available from the corresponding author upon reasonable request.

## Supplementary Materials

The following supporting information can be downloaded at: https://www.mdpi.com/article/10.3390/molecules29050992/s1, Figure S1. Base peak chromatograms in positive and negative ion modes. Table S1. Model parameters for inter-group multivariate statistical analysis. Table S2. Detailed information table for the top 50 differential metabolites.

## Abbreviations

PLP, *Paeonia lactiflora* Pall.; PRA, *Paeoniae Radix* Alba; PRR, *Paeoniae Radix* Rubra; cor, cork; phl, phloem; xyl, xylem; UPLC-MS/MS, UPLC-Q-Exactive-Orbitrap-MS/MS; PCA, principal component analysis; PLS-DA, partial least-squares–discriminant analysis; OPLS-DA, orthogonal partial least-squares–discriminant analysis; QC, quality control; HMDB, the Human Metabolome Database; RPT, response permutation testing; VIP, variable importance in projection; KEGG, Kyoto Encyclopedia of Genes and Genomes.
