# Supplementary figures and images for: Untargeted Metabolomics Based on UPLC-Q-Exactive-Orbitrap-MS/MS Revealed the Differences and Correlations between Different Parts of the Root of Paeonia lactiflora Pall
Source: Molecules. 2024 Feb 24;29(5):992. doi: 10.3390/molecules29050992 (PMC10935087; doi:10.3390/molecules29050992)

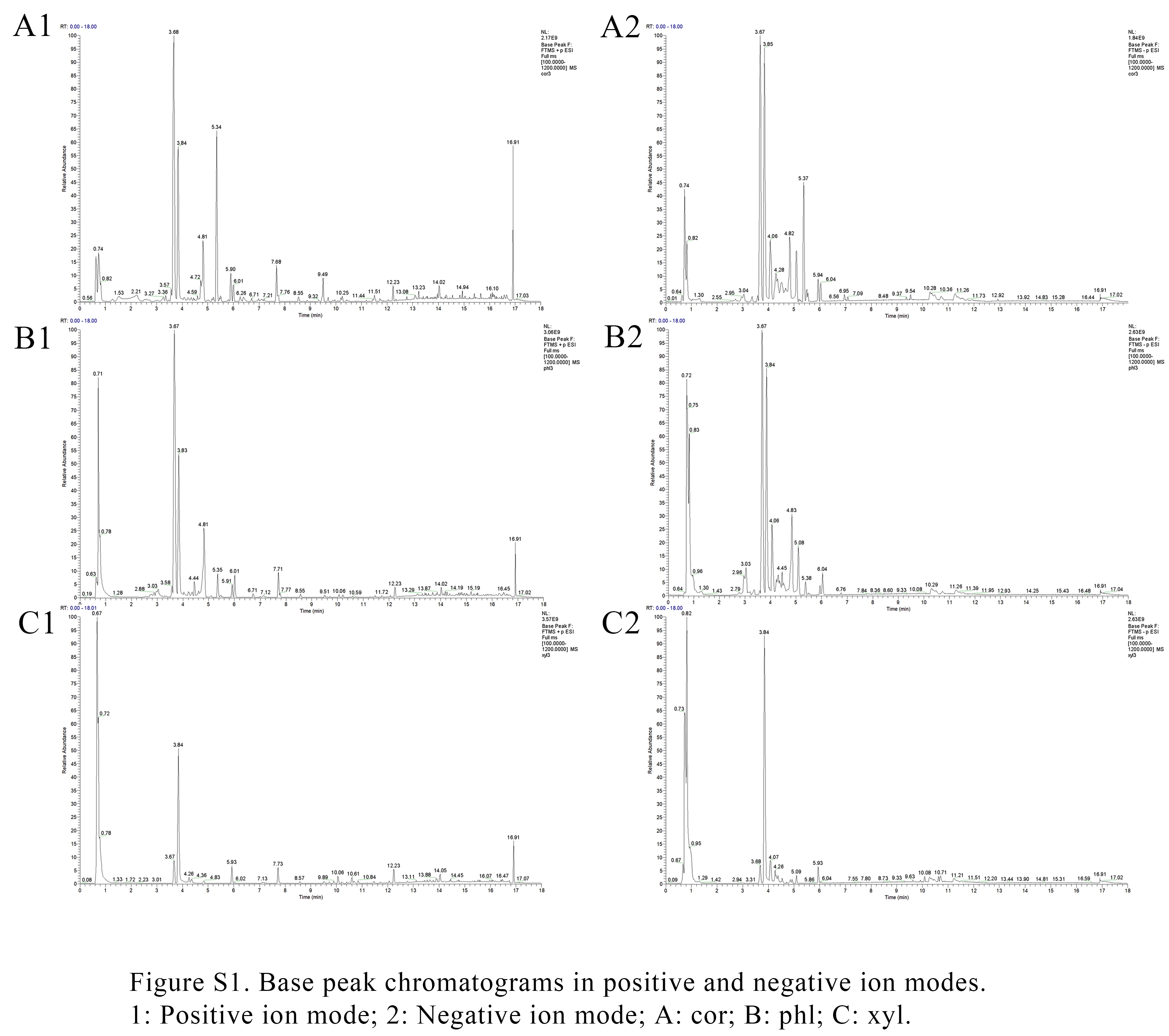

Supplement: Supplementary file 1 [file molecules-29-00992-s001.zip › molecules-2839204-figures.jpg]
